# Comparative Study of Fatty Acid Desaturase (FAD) Members Reveals Their Differential Roles in Upland Cotton

**DOI:** 10.3390/plants14243767

**Published:** 2025-12-10

**Authors:** Fuxin Hu, Shanyu He, Xiangjiang Hou, Jiale He, Panpan Wang, Lei Ma, Di Chen, Haoliang Yan, Juwu Gong, Youlu Yuan, Haihong Shang, Yanpeng Zhao

**Affiliations:** 1 Zhengzhou Research Base, State Key Laboratory of Cotton Bio-Breeding and Integrated Utilization, School of Agriculture and Biomanufacturing, Zhengzhou University, Zhengzhou 450001, China; 2 State Key Laboratory of Cotton Bio-Breeding and Integrated Utilization, Institute of Cotton Research, Chinese Academy of Agricultural Sciences, Anyang 455000, China; 3 Zhengzhou Research Base, State Key Laboratory of Cotton Bio-Breeding and Integrated Utilization, School of Pharmaceutical Sciences, Zhengzhou University, Zhengzhou 450001, China; 4 Kashi Academy of Advanced Agricultural Sciences, Kashi 844000, China

**Keywords:** upland cotton, fatty acid desaturase, FAD2, *GhFAD2-3*, cold stress

## Abstract

Cottonseed oil is rich in unsaturated fatty acids (UFAs), making it suitable for use as edible oil. Fatty acid desaturases (FADs) play a major role in the conversion of monounsaturated fatty acids (MUFAs) to polyunsaturated fatty acids (PUFAs). In total, 39 *GhFAD* genes were detected in upland cotton and divided into five groups in the present study. Gene structure and domain analysis showed that *GhFAD* members within each group were highly conserved. Cis-elements associated with environmental stress and hormone responses were identified in *GhFAD* promoters. The predicted transcription factors and miRNAs targeting these genes suggest extensive roles for *GhFADs* in diverse stress conditions. Analysis of expression profiles indicated that *GhFAD* genes participate extensively in developmental processes and stress adaptation in cotton. Among these, the concurrent high expression of *GhFAD2-1* and low expression of *GhFAD3* are consistent with the typical fatty acid profile of cottonseed oil. *GhFAD3-2* and *GhFAD3-1* exhibit a complementary expression profiles, suggesting they may operate in a relay manner during fiber development. Additionally, experimental evidence established that *GhFAD2-3* is involved in the cold stress response. This research delivers a thorough characterization of the *GhFAD* genes in upland cotton, thereby establishing a solid groundwork for future functional genomics studies.

## 1. Introduction

Cotton (*Gossypium* spp.) is the most important source of natural fiber, along with a vital provider of edible oil for human and protein for livestock. Upland cotton (*G. hirsutum*) accounts for over 95 percent of cotton cultivation, owing to its prominent fiber yield and environmental adaptability [1]. Cottonseed composition is approximately 16% oil, 45% meal, 25% hull, and 8% fuzz [2]. What is more, cottonseed oil ranks as the fifth most produced edible oil worldwide [3]. It is rich in unsaturated fatty acids, primarily linoleic acid (C18:2, 58%) and oleic acid (C18:1, 15%) [4]. Notably, oils rich in unsaturated fatty acids are considered desirable dietary components, because their consumption is linked to multiple health benefits [5,6].

Desaturation reactions, during which saturated fatty acids (SFAs) are converted into unsaturated fatty acids (UFAs), are catalyzed by enzymes encoded by stearoyl-ACP desaturase (*SAD*) and fatty acid desaturase (*FAD*) genes. Specifically, SAD enzymes catalyze the conversion of stearic acid (C18:0) to oleic acid (C18:1), a step that typically occurs before fatty acids are incorporated into glycerolipids [7]. In contrast, FADs encode membrane-bound desaturases that introduce double bonds into fatty acyl chains. Studies on Arabidopsis mutants have revealed the involvement of multiple FAD genes in this process, including *FAD2*, *FAD3*, *FAD4*, *FAD5*, *FAD6*, *FAD7*, and *FAD8* [8]. Notably, FAD enzymes exhibit distinct substrate specificities. FAD4 and FAD5 desaturases primarily target 16-carbon SFAs, whereas FAD2 and FAD6 act on 18-carbon monounsaturated fatty acids (MUFAs) to produce C18:2; hence, these are classified as ω-6 FAD genes [9]. Meanwhile, FAD3, FAD7, and FAD8 catalyze the conversion of C18:2 to C18:3 and are thus termed ω-3 FAD genes [10]. Subcellular localization studies indicate that FAD2 and FAD3 reside on the endoplasmic reticulum (ER) membrane and are primarily responsible for UFA synthesis in all membrane lipids except those in plastids. In contrast, FAD4, FAD5, FAD6, FAD7, and FAD8 are plastid membrane-localized and participate in the further desaturation of plastid membrane lipids [11].

The functions of FAD genes have been extensively characterized across multiple plant species. In soybean, two novel FAD2 members, FAD2-2B and FAD2-2C, function as key regulators of C18:2 levels during pod development under colder climates [12]. In rapeseed, knockout of *FAD2* genes generated *FAD2-1A* and *FAD2-1B* mutants, resulting in an increase in C18:1 content from 20% to 80% and a reduction in C18:2 from 50% to below 4% [13]. Similarly, seed-specific simultaneous suppression of *FAD2.2* and *FATB* in safflower led to ultra-high C18:1 content and enhanced oxidative stability [14]. EMS mutagenesis in rapeseed identified mutants with elevated C18:1 content, where sequence variations were detected in *FAD2* and *ROD* genes [15]. In sea-island cotton (*G. barbadense*), the *GbFAD2-1D* mutant was characterized; its transient expression in Arabidopsis leaves significantly reduced C18:2 content [16]. Subsequent studies revealed that while the missing C-terminal amino acid residues in FAD2-1D were not absolutely essential for enzymatic activity, they were critical for proper endoplasmic reticulum membrane localization [17]. In upland cotton, silencing *GhFAD2* expression increased C18:1 level from 15% to 77% [18], while simultaneous suppression of *GhFATB* and *GhFAD2-1* substantially elevated C18:1 content while reducing palmitic acid (C16:0) and C18:2 levels [19]. Additionally, gene editing of *GhFAD2-1* successfully generated high-oleic cotton [3]. Similarly, high-oleic peanuts were developed by knocking out *AhFAD2* homologs [20]. Beyond lipid metabolism, FAD genes also participate in plant development. Single-cell sequencing analysis revealed that *FAD2* mutants inhibit leaf growth in peanuts [21]. In carrots, the *DcFAD2* hub gene is essential for falcarindiol biosynthesis, and the expansion of the *DcFAD2* gene family underlies falcarindiol enrichment [22]. In upland cotton, *GhFAD2-3* was reported to be involved in pollen development [23], while *GhFAD3-4* promotes fiber elongation and cell wall thickening by enhancing phosphatidylinositol (PI) and inositol trisphosphate (IP3) accumulation [24].

FADs are widely involved in plant environmental adaptation. In upland cotton, the expression of the FAD2 gene is regulated by low temperature and light [25]. High temperatures inhibit ω-6 and ω-3 desaturation in phosphatidylcholine; notably, ω-6 desaturation is primarily regulated by cis-acting sequence variations in the intronic region within the 5′ UTR of the *FAD2* gene, which determine its expression levels [26]. In olives, microsomal FAD activity is not only involved in fruit development but also induced by water availability, temperature, light, and mechanical damage [27]. Similarly, low temperature and salt stress affect *FAD2* gene expression and alter fatty acid composition in safflower [28]. Furthermore, the degree of polyunsaturation in endoplasmic reticulum (ER) glycolipids influences ER stress sensitivity. *FAD2* mutants exhibit heightened sensitivity to tunicamycin, indicating that the C18:1 to C18:2 ratio is crucial role for ER-mediated stress responses [29]. Beyond fatty acid metabolism, *FAD* genes also participate in biotic stress responses. For instance, silencing *SlFAD2-7* expression in tomatoes leads to a significant increase in aphid infestation [30], underscoring the role of FAD enzymes in enhancing plant resistance through the modulation of multiple lipid metabolic pathways [31]. In rice, the viral protein P10 binds to OsFAD7 and promotes its degradation, thereby reducing the synthesis of jasmonic acid (JA) precursor to enhance viral infectivity [32].

The identification of fatty acid desaturase (FAD) genes in plant genomes is crucial for understanding their evolutionary history and functional diversity. A total of 20 members were identified in rice [33], and family-wide analysis in maize revealed their involvement in cold and heat stress responses [34]. Transcriptome-based analysis in walnut uncovered 24 *FAD* genes, with JrFAD3-1 potentially participating in polyunsaturated fatty acid biosynthesis [35]. Investigation of the FAD family in sage (*Salvia*) demonstrated its roles in cold stress adaptation and oil formation [36]. Similarly, analysis of membrane-bound FADs in faba bean suggested that FAD2-1 may contribute to oil synthesis, while the low expression of *CeFAD3* likely accounts for the reduced linolenic acid content [37]. Furthermore, a total of 43 *LuFAD* genes were identified in flax, with LuFAD2.1 implicated in cold stress response [38]. In cotton, 19 *FAD* members were identified in *G. raimondii*, and 39 membrane-associated FADs were characterized in upland cotton, highlighting their potential roles in cold stress [39,40].

However, the comprehensive characterization and functional validation of FAD genes in upland cotton have not yet been simultaneously conducted. With advancements in the improve of genome quality and the completion of telomere-to-telomere (T2T) genome assemblies, it is essential to perform thorough analyses of *FAD* gene families in upland cotton. To characterize the fundamental features of *FAD* genes in cotton, this study conducted a comprehensive comparative analysis of *FAD* genes in upland cotton based on the T2T genome [41]. Gene structure, the conserve domains and the cis-elements in promoter were investigated. Meanwhile, the expression patterns of *GhFADs* in various developmental stages and abiotic stresses were analyzed and the regulatory relationships with transcription factors (TFs) and miRNA were explored. Furthermore, the function of *GhFAD2-3* was examined in the present study. This study will provide a foundation for the utilization of *FAD* genes in molecular breeding programs.

## 2. Results

### 2.1. Identification of FAD Gene Family in Gossypium

**Figure 1:**
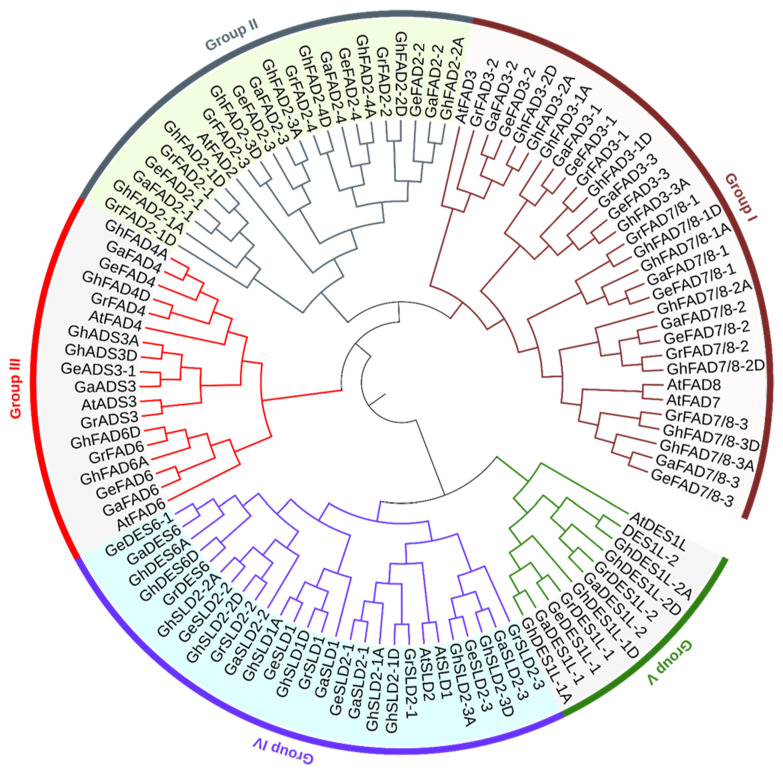
Phylogenetic analysis of FAD protein family in *Gossypium*. The phylogenetic tree was constructed using MEGA-12 and visualized with iTOL (v7) software. Protein sequences from *G. hirsutum* (Gh), *G. herbaceum* (Gher), *G. arboreum* (Ga), and *G. raimondii* (Gr) are included.

The fatty acid desaturase (FAD) domain (PF00487) was employed as a query to identify FAD genes in cotton genomes using hmmsearch against their protein sequences. This analysis identified 20, 20, 20, and 39 FAD genes in the A1, A2, D5, and AD1 genomes, respectively (Table 1 and Table S2). Phylogenetic analysis revealed that these 99 FAD genes are classified into five distinct groups: Group I comprises FAD3 and FAD7/8; Group II forms a separate cluster containing FAD2 genes; Group III includes FAD4, ADS3, and FAD6; Group IV contains SLD1, SLD2, and DES6; and Group V consists of DES1L genes. Furthermore, orthologous FAD genes from the different cotton genomes consistently clustered together within the same group, demonstrating strong evolutionary conservation (Figure 1). Notably, FAD3-3 was present in the A genomes (A1 and A2) and the At subgenome of AD1, but absent from the D5 genome and the Dt subgenome of AD1. Additionally, while two GrFAD2-1 genes were identified in the D5 genome, only one ortholog (*GhFAD2-1D*) was detected in the Dt subgenome, suggesting the loss of one GrFAD2-1 gene during interspecific hybridization and genome polyploidization.

In upland cotton (AD1), the 39 FAD genes were classified into FAD2, FAD3, FAD4, FAD6, FAD7/8, ADS3, DES6, SLD1, SLD2, and DES1L subgroups based on sequence homology with *A. thaliana* FAD genes. The coding sequence (CDS) length ranged from 927 bp (*GhFAD3-3A*) to 1353 bp (*GhFAD7/8-3A* and *GhFAD7/8-3D*). Characterization of the deduced amino acid sequences showed that the molecular weight (MW) ranged from 34.51 kDa (GhFAD4D) to 51.18 kDa (GhFAD7/8-3A), and the theoretical isoelectric point (pI) varied from 6.95 (GhDES1L-1D) to 9.37 (GhADS3D). Subcellular localization predictions using WoLF PSORT indicated that 15 GhFAD proteins are likely localized to the plastid, while four are predicted to reside in the endoplasmic reticulum (ER) (Table 1).

### 2.2. Conserved Structural Features Analysis of GhFAD Members

**Figure 2:**
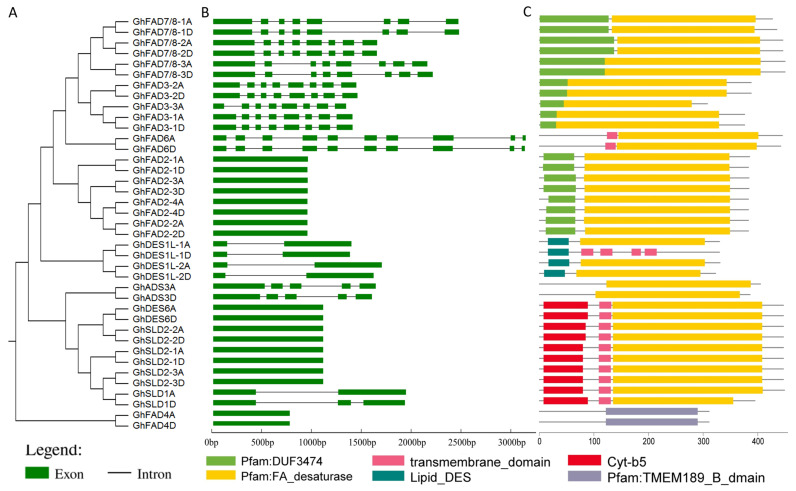
Gene structure and conserved domains of *GhFAD* members. (**A**) Phylogenetic tree of *GhFAD* genes. (**B**) Exon–intron structure of *GhFAD* genes. (**C**) Conserved protein domains identified in *GhFAD* proteins.

As upland cotton represents the predominant cultivated species in terms of both planting area and fiber production, a detailed analysis of the *FAD* gene family in upland cotton was undertaken in this research. A total of 39 GhFAD members were analyzed to investigate their phylogenetic relationships and structures. The results indicated that the phylogenetic clustering of *GhFAD* members was consistent with that observed in other cotton species (Figure 2A). Analysis of gene organization revealed that the number of exons in *GhFAD* genes varied between 1 and 10. Specifically, *GhDES6*, *GhFAD2*, *GhFAD4*, and *GhSLD2* genes were intronless, while *GhDES1L* and *GhSLD* genes typically contained two exons, *GhADS* genes had five exons, *GhFAD3* and *GhFAD7/8* possessed eight exons, and *GhFAD6* contained the highest number of exons (10) (Figure 2B). Furthermore, conserved domain analysis showed that all GhFAD proteins contained a fatty acid desaturase domain, except for GhDES1L-1D, GhFAD4A, and GhFAD4D. Characteristically, GhFAD3, GhFAD7/8, and GhFAD2 proteins contained the DUF3474 domain; GhDES1L proteins possessed a lipid_DES domain; GhDES6, GhSLD1, and GhSLD2 members contained a Cyt-b5 domain; and GhFAD4s featured a TMEM189_B domain. Predictions indicated that transmembrane domain only present in GhFAD6, GhDES6, GhSLD1, GhSLD2, and *GhDES1L-1D* proteins (Figure 2C). Additionally, the ten most conserved motifs were identified among GhFAD members. GhFAD proteins within the same clade generally contain similar motif compositions. All GhFAD members possess motif 2, with the exception of GhFAD4A and GhFAD4D proteins. In particular, members of the GhFAD2, GhFAD3, and GhFAD7/8 subgroups contain motif 1, motif 4, motif 5, motif 6, and motif 7. GhFAD6 contains motif 1. GhSLD2 and *GhDES6* members share motif 3, motif 8, motif 9, and motif 10, whereas GhSLD1 lacks motif 9 (Figure S1). The conservation of motifs suggests that GhFAD members within the same clade may perform similar biological functions.

### 2.3. Chromosomal Localization and Gene Synteny Analysis of GhFAD Genes

**Figure 3:**
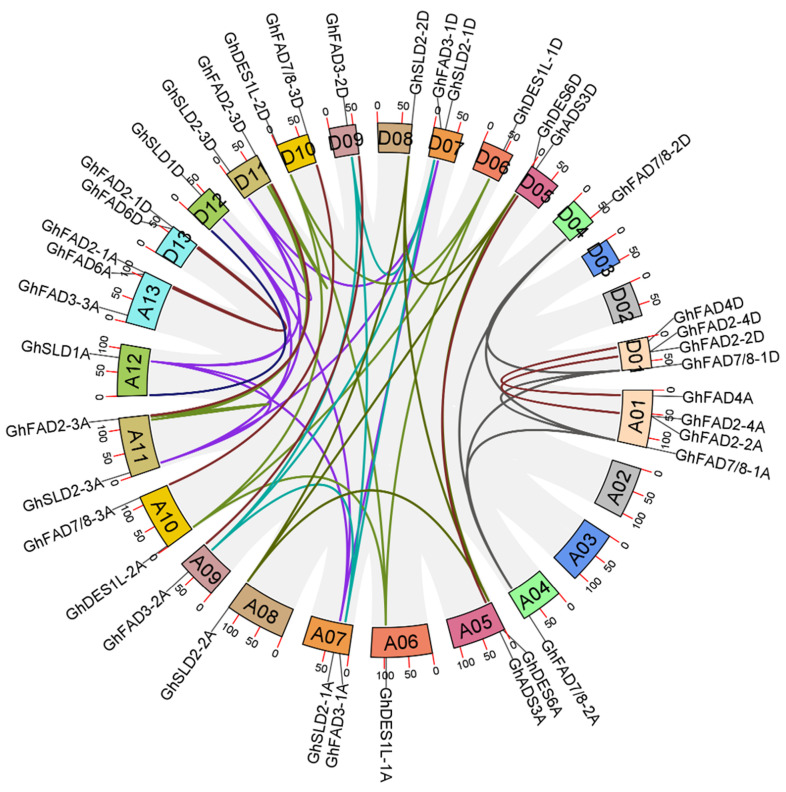
Synteny analysis of *GhFAD* genes in upland cotton. Homologous gene pairs with a one-to-one syntenic relationship between the At and Dt subgenomes are highlighted in brown. Those with many-to-many relationships are shown in different, yet consistent, colors.

To elucidate the homologous relationships among *GhFAD* genes, we performed chromosomal localization and gene synteny analysis. Chromosomal localization revealed that most *GhFAD* loci were highly conserved between the At and Dt subgenomes. The number and distribution of *GhFAD* genes on chromosomes in the At subgenome were closely mirrored those on their homologous chromosomes in the Dt subgenome. An exception was *GhFAD3-3A*, which lacked a homologous counterpart in the Dt subgenome (Figure S2). Gene synteny analysis further revealed frequent duplication events among *GhFADs* during cotton evolution, with 51 pairs of collinear relationships were found among the 39 *GhFAD* genes. Based on the location of the duplicated genes, it was found that fragmental duplication mainly led to the expansion of *GhFAD* members in upland cotton (Figure 3).

### 2.4. Analysis of Cis-Elements in GhFAD Promoter and Regulatory Relationships

**Figure 4:**
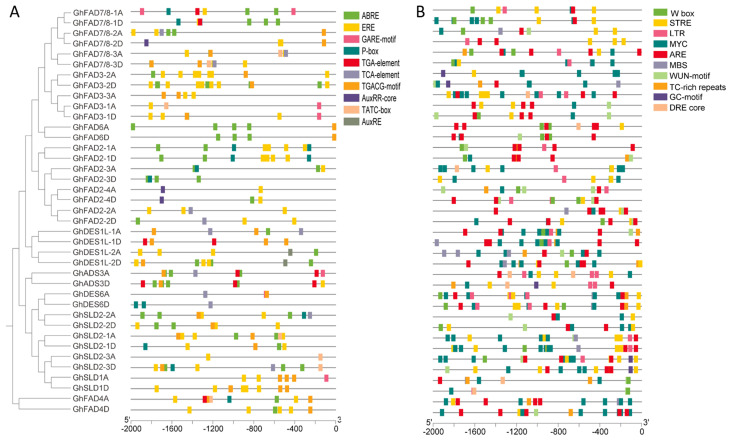
Cis-acting regulatory elements in the promoter regions of *GhFAD* genes. (**A**) Hormone-related cis-elements. (**B**) Environmental response-related cis elements.

To investigate the cis-acting regulatory elements in *GhFAD* genes, a 2 kb sequence upstream of the start codon (ATG) of each *GhFAD* gene was analyzed using the PlantCARE database. A total of 93 kinds of distinct cis-elements were detected in of the 39 *GhFAD* promoter regions (Table S3). Among these, numerous elements were associated with light responsiveness. Notably, several elements involved in environmental stress and hormone response were prominently identified. Ten environmental stress-related elements were detected, with a predominance of those related to drought stress (MYC) and general stress response (STRE). Among the hormone-responsive elements, ERE, ABRE, and CGTCA-motif cis-elements were more abundant, suggesting that *GhFAD* genes may be primarily responsive to ethylene, abscisic acid, and methyl jasmonate (MeJA), respectively (Figure 4).

**Figure 5:**
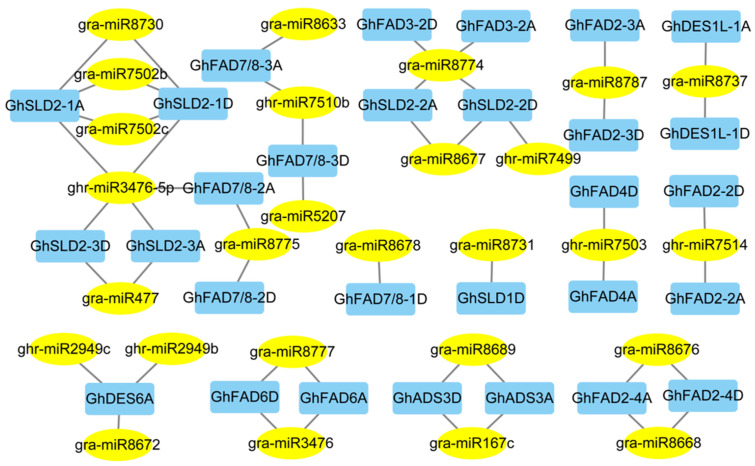
Regulatory networks of miRNAs targeting *GhFAD* genes. miRNAs are depicted as yellow ovals, while target *GhFAD* genes are depicted as blue rectangles.

Cis-elements function by binding transcription factors (TFs) to regulate the initiation of gene expression. Hence, the potential TFs targeting the *GhFAD* genes were predicted. In total, 568 regulatory relationships were detected. The analysis revealed that *GhADS3* homologous genes are potentially regulated by a greater number of TFs, including well-known stress-related TFs such as ERF, Dof, and MYB. Furthermore, many *GhFAD* genes are likely regulated by Dof, ERF, and NAC TFs, suggesting their roles in plant development and stress responses (Figure S3). In addition to transcription factors, miRNAs are also widely recognized as key regulators of gene expression, playing crucial roles in abiotic stress responses. To explore the potential post-transcriptional regulation of *GhFAD* genes, 29 *GhFAD* genes were supposed regulated by 27 putative miRNAs, encompassing 37 interaction pairs (Table S4). Notably, *GhSLD2-1A* and *GhSLD2-1D* were the most frequently targeted genes, each interacting with four different miRNAs. *GhDES6A* and *GhSLD2-2D* were each predicted to be regulated by three miRNAs, while gra-miR8774 was found to target four *GhFAD* genes (Figure 5). Notably, most homologous *GhFAD* gene pairs were predicted to be regulated by the same miRNAs, suggesting conserved functional roles between subgenomes. These predictions provide a foundation for future experimental studies on miRNA-mediated regulation of *GhFAD* genes.

### 2.5. Expression Profiling of GhFAD Genes in Upland Cotton

**Figure 6:**
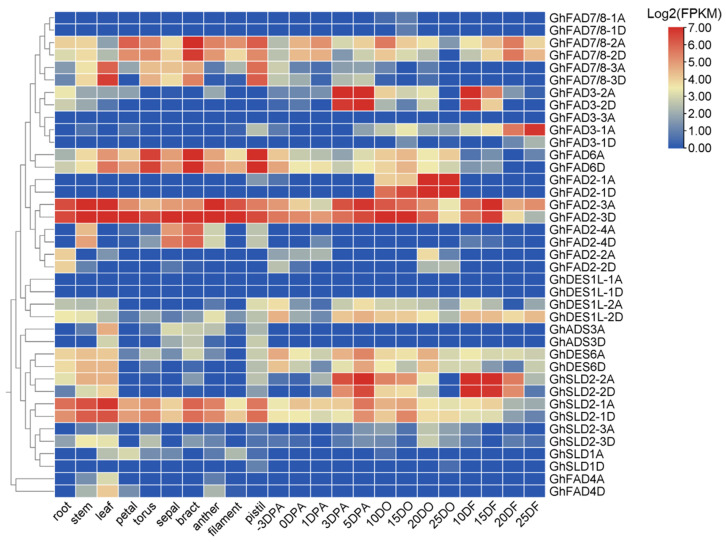
Expression profiles of *GhFAD* genes in different tissues of upland cotton. DO, days post-anthesis (DPA) ovule; DF, DPA fiber.

Publicly available expression datasets for upland cotton were analyzed to investigate the expression patterns of *GhFAD* genes across different tissues and at various stages of ovule and fiber development (Figure 6). The analysis revealed that many *GhFAD* genes were barely expressed, including homologous pairs of *GhFAD7/8-1*, *GhDES1L-1*, *GhSLD1*, and *GhFAD4*. In contrast, *GhFAD7/8-2*, *GhFAD2-3*, *GhFAD6*, and *GhSLD2-1* homologous genes were abundantly expressed. Notably, *GhFAD3-2A/D* and *GhSLD2-2A/D* genes were highly expressed at 3 DO, 5 DO, 10 DF, and 15DF, suggesting a potential role in fiber elongation. Conversely, *GhFAD3-1A* was preferentially expressed at later stages (20 and 25 DPA), displaying a complementary expression pattern to *GhFAD3-2* during fiber development. *GhFAD6* genes were abundantly expressed in reproductive organs, including the torus, bract, and pistil. A key finding was the specific and preferential expression of *GhFAD2-1* homologous genes during the oil accumulation phase in cottonseeds (10, 15, 20, and 25 DPA), indicating a likely role for *GhFAD2-1* in fatty acid synthesis. Furthermore, while *FAD2-3A/D* was highly expressed across all examined tissues, *FAD2-2A/D* and *FAD2-4A/D* showed consistently low expression levels (Figure 6). These divergent expression profiles among gene duplicates suggest that functional specialization has occurred during cotton evolution.

### 2.6. Response of GhFAD Genes to Abiotic Stresses in Upland Cotton

**Figure 7:**
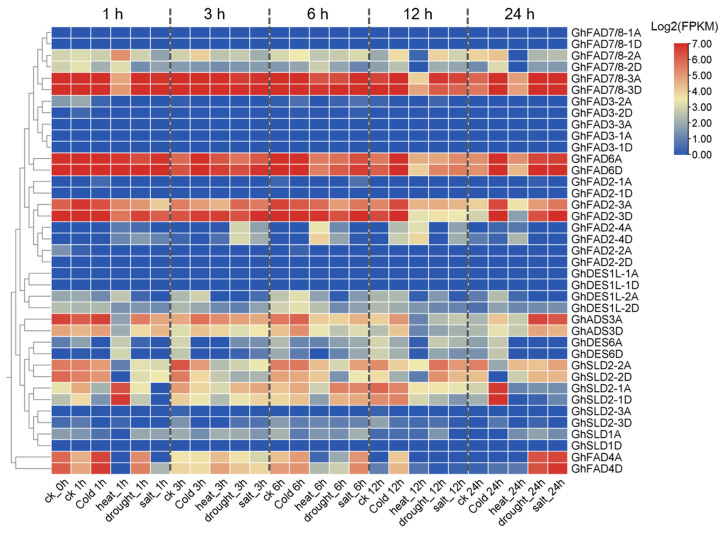
Expression profiles of *GhFAD* genes under abiotic stress conditions. Cold, 4 °C; heat, 37 °C; drought, 20% PEG6000; salt, 20% NaCl.

Expression profiles of *GhFAD* genes under various abiotic stresses—including cold, heat, drought, and salt treatments across multiple time points—were analyzed using publicly available transcriptome data (Figure 7). Genes with low basal expression levels in leaves, such as *GhFAD7/8-1*, *GhFAD3*, *GhFAD2-1*, *GhFAD2-2*, and *GhDES1L*, showed no significant response to the applied stresses. By contrast, highly expressed genes including *GhFAD7/8-3*, *GhFAD6*, and *GhFAD2-3* exhibited distinct expression changes under stress conditions (Figure 7. The expression of *GhFAD7/8-3* genes was down-regulated under heat stress at 1, 12, and 24 hours (h). *GhFAD6* genes were up-regulated at 12 and 24 h under cold stress. *GhFAD2-3* genes were significantly up-regulated at 12 and 24 h under cold stress, but showed high expression only at 24 h under drought and salt conditions (Figure 7). *GhSLD2-1* was induced expression at 1 h of heat stress and 24 h of cold stress. Additionally, *GhFAD4* genes were dramatically induced under drought and salt stresses at 24 h (Figure 7). These expression patterns are supported by the presence of corresponding stress-related cis-elements, such as LTR, MYB, and MYC motifs, in the promoter regions of the respective *GhFAD* genes, indicating a transcriptional regulatory basis for their stress-responsive expression.

**Figure 8:**
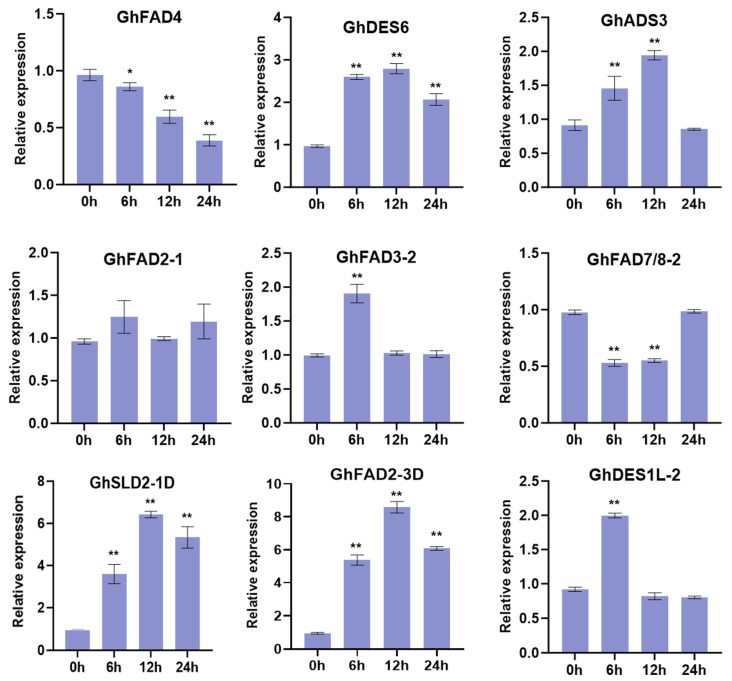
Expression levels of *GhFAD* genes under cold stress. *, *p* < 0.05; **, *p* < 0.01.

Meanwhile, the expression patterns of nine *GhFAD* genes respond to cold stress were examined by RT-qPCR. The relative expression level of *GhFAD* genes in cotton leaves at 0, 6, 12, and 24 h were measured (Figure 8). Under cold stress, eight *GhFAD* genes were significantly up- or down-regulated, with the exception of *GhFAD2-1*. The expression level of *GhFAD4* decreased continuously, while *GhFAD7/8-2* was down-regulated at 6 and 12 h after cold exposure. In contrast, *GhFAD3-2* and *GhDES1L-2* were up-regulated at 3 h, and *GhADS* expression was induced at 3 and 6 h. Meanwhile, *GhDES6*, *GhSLD2-1*, and *GhFAD2-3* showed continuous induction of expression, reaching their peak levels at 12 h. Notably, *GhFAD2-3* was markedly induced, with expression levels 5.6-, 9.0-, and 6.4-fold higher at 6, 12, and 24 h, respectively, compared to the 0 h control (Figure 8).

### 2.7. Overexpression of GhFAD2-3 Enhances Cold Stress Tolerance in Arabidopsis

**Figure 9:**
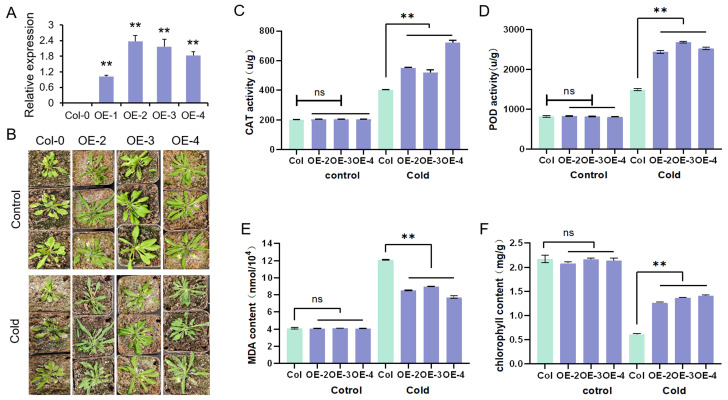
Overexpression of *GhFAD2-3* enhances cold tolerance in Arabidopsis. (**A**) RT-qPCR analysis of *GhFAD2-3* expression in OE lines. (**B**) Phenotypic responses to cold stress in *GhFAD2-3*-overexpressing and wild-type *Arabidopsis* plants. (**C**–**F**) Statistical analysis of physiological parameters of CAT and POD activities, and MDA and chlorophyll contents. ns, *p* > 0.05; **, *p* < 0.01.

Given the induced expression of *GhFAD2-3* under cold stress, its biological function was further investigated through heterologous overexpression in *Arabidopsis*. RT-qPCR analysis confirmed that *GhFAD2-3* was expressed in transgenic *Arabidopsis* lines (Figure 9A). Homozygous T_3_-generation lines were selected and subjected to cold stress treatment at 4 °C. After 24 h of cold exposure, leaves of wild-type Arabidopsis plants exhibited obvious wilting, whereas the overexpression lines showed substantially reduced phenotypic damage (Figure 9B). Measurements of physiological parameter further supported the enhanced cold tolerance of transgenic plants. To investigate the role of *GhFAD2-3D* in reducing reactive oxygen species (ROS accumulation), we evaluated the activities of the antioxidant enzymes catalase (CAT) and peroxidase (POD). Under normal conditions, the activities of CAT and POD did not differ significantly between wild-type (WT) and transgenic plants. Following cold stress, however, the activities of both enzymes were significantly higher in the transgenic lines (Figure 9C,D). Furthermore, under cold stress, the *GhFAD2-3D*-overexpressing plants displayed enhanced chlorophyll retention and reduced malondialdehyde (MDA) accumulation compared to the WT controls (Figure 9E,F). Collectively, these results demonstrate that overexpression of *GhFAD2-3* significantly enhances cold stress tolerance in *Arabidopsis*.

### 2.8. Functional Validation of GhFAD2-3 in Cold Stress Response via VIGS in Upland Cotton

**Figure 10:**
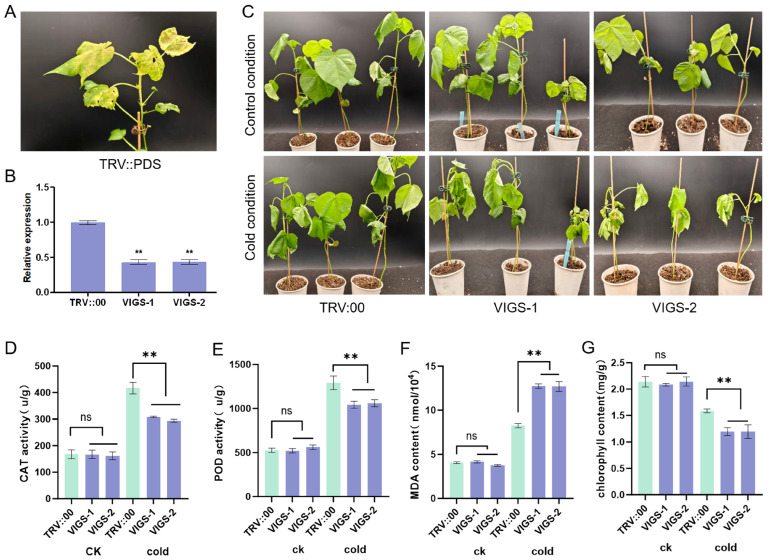
Functional validation of *GhFAD2-3* in cold stress response in cotton. (**A**) The mottled leaves phenotype of the positive control line (TRV::PDS). (**B**) Expression level of *GhFAD2-3* in TRV::GhFAD2-3 and TRV::00 cotton plants. (**C**) Chilling injury phenotype under control and cold stress. (**D**–**G**) Statistical analysis of physiological indices, including CAT and POD activities, and MDA and chlorophyll contents. ns, *p* > 0.05; **, *p* < 0.01.

To further elucidate the functional role of *GhFAD2-3* in its native context, we employed virus-induced gene silencing (VIGS) to downregulate *GhFAD2-3* expression in upland cotton. When the mottled leaf phenotype appeared in the positive control line (TRV::PDS), RT-qPCR analysis confirmed a significant reduction in *GhFAD2-3* transcript levels in TRV::GhFAD2-3 plants (VIGS-1 and VIGS-2) compared to the empty vector control (TRV::00) (Figure 10A,B). When subjected to cold stress at 10 °C for 24 h, the TRV::GhFAD2-3 plants exhibited more severe wilting than the control plants (Figure 10C). Physiological assessments further demonstrated that the silencing of *GhFAD2-3* significantly impaired CAT and POD activities, promoted chlorophyll degradation, and increased malondialdehyde (MDA) content under cold stress conditions (Figure 10D–F). Taken together, these results demonstrate that knockdown of *GhFAD2-3* compromises cold tolerance in cotton and perturbs the expression of core cold-responsive regulators, confirming the essential role of *GhFAD2-3* in the low-temperature stress response pathway.

## 3. Discussion

### 3.1. Functional Differentiation of FAD2 Genes During Evolution

Extensive studies have revealed significant functional differentiation of FAD2 genes throughout plant evolution. While Arabidopsis contains only a single *FAD2* gene participating in both seed oil biosynthesis and membrane lipid formation [8], most other plants possess FAD2 multigene families comprising 3 to 11 members. Soybean contains five *FAD2* genes, with *FAD2-1A* and *FAD2-1B* primarily regulating oleic acid desaturation in seeds [13]. Olive (*Olea europaea*) harbors five *FAD2* genes, among which *OeFAD2-1* functions specifically in seed oil biosynthesis, *OeFAD2-2* shows broad expression across tissues, and *OeFAD2-2* along with *OeFAD2-5* play crucial roles in linoleic acid production in the mesocarp [27]. Safflower (*Carthamus tinctorius*) possesses a notably expanded FAD2 family of eleven members, with *FAD2-1* demonstrating seed-specific expression and *FAD2-2* serving as a constitutively expressed housekeeping gene [14]. Similarly, sunflower (*Helianthus annuus*) exhibits FAD2 family expansion, where *CarFAD2-12* shows tissue-specific high expression in seeds while other duplicates are predominantly expressed in ovaries [42].

Our investigation identified eight FAD2 members in cotton that have similarly undergone functional specialization. *GhFAD2-1A* and *GhFAD2-1D*, identified as seed-type genes, exhibit seed-specific expression patterns (Figure 6), consistent with their conserved role in converting C18:1 to C18:2. In contrast, *GhFAD2-2A/D* shows exclusive expression in roots and 20-day-old ovules, *GhFAD2-4A/D* is preferential expressed in stems, sepals, and bracts, while *GhFAD2-3A/D* maintains constitutive expression across tissues (Figure 6). This differential expression pattern demonstrates clearly functional divergence among cotton FAD2 paralogs. The expansion and functional differentiation of the FAD2 gene family in cotton represents a classic example of evolutionary adaptation following polyploidization. Derived from common ancestral genes, the eight *GhFAD2* members have undergone subfunctionalization and/or neofunctionalization, partitioning their original biological roles. This is evidenced by the conserved function of *GhFAD2-1A/D* in seed-specific oleic acid desaturation, while paralogs such as *GhFAD2-2A/D* and *GhFAD2-3A/D* have acquired novel expression patterns, likely fine-tuning membrane lipid homeostasis in roots or supporting lipid requirements for rapid growth across various tissues. This genetic innovation through gene family expansion provides cotton with enhanced regulatory flexibility to optimize lipid composition for diverse physiological needs, ranging from seed storage to environmental adaptation.

### 3.2. Fatty Acid Composition Regulated by FAD2 and FAD3

The ratio of oleic acid (C18:1) to linoleic acid (C18:2) is critical determinant of the nutritional quality and oxidative stability of plant-derived edible oils [5]. This ratio is primarily regulated by the synergistic actions of two key enzymes: fatty acid desaturases FAD2 (oleate desaturase) and FAD3 (linoleate desaturase). FAD2 catalyzes the conversion of C18:1 to C18:2, while FAD3 further desaturates C18:2 to α-linolenic acid (C18:3) in extra-plastidial compartments [4]. In *Arabidopsis dgat1* mutants, FAD2 is essential to provide polyunsaturated fatty acid (PUFA) substrates for normal seed development [43]. The creation of high-oleic peanut varieties by disrupting *AhFAD2A/B* genes demonstrates the critical role of FAD2 in determining fatty acid composition without compromising agronomic traits [20]. Meanwhile, FAD3 is more effective enzyme that converts C18:2 to C18:3 in extra-plastid [10]. Meanwhile, FAD3 serves as the more effective enzyme for converting C18:2 to C18:3 in extra-plastidial compartments [37]. Genetic engineering approaches co-expressing *AtFAD2sm* and *BnFAD3* while eliminating *FAE1* have successfully achieved ultra-high C18:3 contents in Arabidopsis and camelina [44]. In cotton, silencing *GhFAD2* expression elevated oleic acid levels from 15% to 77% [18], while simultaneous suppression of *GhFatB* and *GhFAD2* substantially increased oleic acid content while reducing palmitic and linoleic acid levels [19]. More recently, gene editing of *GhFAD2-1* successfully generated high-C18:1 cotton [3], and the identification of LncRNA pseudo-GhFAD2-1 regulating oil composition and seed size further highlights the regulatory complexity of this pathway [45].

In our study, the seed-specific high expression of *GhFAD2-1A* and *GhFAD2-1D* confirms their primary role in converting C18:1 to C18:2 during cottonseed development. The coordinated action of FAD2 and FAD3 fundamentally determines the final composition of polyunsaturated fatty acids in plants (Figure 6). Cottonseed oil is characterized by high linoleic acid content (>55%) but minimal linolenic acid (0.3%) [4]. Our expression analysis provides the molecular basis for this characteristic profile: the strong expression of *GhFAD2-1* ensures abundant linoleic acid production, while the consistently low expression of *GhFAD3* genes throughout seed development creates a metabolic bottleneck, limiting the conversion to linolenic acid. This mechanistic interplay suggests that the balance between FAD2 and FAD3 activities serves as the critical determinant for the C18:2/C18:3 ratio.

### 3.3. GhFAD3-1 and GhFAD3-2 Sequentially Regulate Fiber Development

Emerging evidence indicates that *FAD3* genes play crucial roles in cotton fiber development beyond their canonical function in fatty acid desaturation. Previous studies have established that *FAD3* silencing results in shorter cotton fibers [46], while heterologous expression of rapeseed *BnFAD3* enhances fiber length [47]. More recently, *GhFAD3-4* has been shown to promote both fiber elongation and cell wall thickening through enhanced accumulation of phosphatidylinositol (PI) and inositol trisphosphate (IP3) [24]. In this study, we identified five *GhFAD3* genes, with *FAD3-3* being exclusively present in the At subgenome (Table 1). Expression profiling revealed substantial functional divergence among these GhFAD3 members. *GhFAD3-3* appears to be transcriptionally silent, whereas *GhFAD3-2A/D* exhibit specific, high expression during early ovule (3 DPA) and fiber development stages (10–15 DPA), consistent with a primary role in fiber elongation (Figure 6). Conversely, *GhFAD3-1A* is specifically upregulated during later fiber development (20–25 DPA), suggesting its involvement in secondary wall thickening. The expression dominance of *GhFAD3-1A* over its homeolog *GhFAD3-1D* aligns with the established evolutionary pattern of At subgenome predominance in fiber development [48].

Based on these distinct temporal expression patterns, we propose a relay regulation model wherein *GhFAD3-2* and *GhFAD3-1* coordinately regulate cotton fiber development through stage-specific actions. In this model, *GhFAD3-2* serves as an early-phase regulator driving fiber elongation, while *GhFAD3-1* functions as a late-phase regulator promoting secondary wall deposition. This temporal specialization exemplifies how gene duplication and functional divergence can optimize complex developmental processes.

### 3.4. GhFAD2-3 Was Involved in Cold Stress Response

Accumulating evidence from diverse plant species underscores the crucial roles of *FAD2* genes in cold stress adaptation. In safflower, low temperature significantly influences *FAD2* expression and alters fatty acid composition [28]. Family-wide analysis of *FAD* genes in maize revealed their collective involvement in cold stress responses [34], while similar investigations in sage (*Salvia*) demonstrated FAD family participation in cold adaptation [36]. Notably, among 43 *LuFAD* genes identified in flax, overexpression of *LuFAD2.1* enhanced cold tolerance in Arabidopsis [38]. In cotton, *FAD2* expression is known to be regulated by low temperature [25], and *FAD7/8-1* is specifically induced under cold stress [49]. Multiple *FAD* genes responsive to cold stress have been identified in both *G. raimondii* and upland cotton [39,40], collectively suggesting conserved functions of *FAD* genes in cold stress adaptation. In this study, we identified several *GhFAD* genes responsive to cold stress, such as GhFAD4, *GhSLD2-1*, *GhFAD6*, and *GhFAD2-3* (Figure 7). RT-qPCR validation confirmed that cold stress induced the expression of multiple genes, among which *GhDES6*, *GhADS3*, *GhSLD2-1D*, and *GhFAD2-3D* were all significantly up-regulated (Figure 8).

While *GhFAD2-3* has been previously reported to participate in pollen development in upland cotton [23], our study reveals its additional role in cold stress response. We observed significant up-regulation of *GhFAD2-3A/D* genes after 12 and 24 h of cold treatment (Figure 7), consistent with the presence of low-temperature responsive (LTR) cis-elements in their promoter regions (Figure 4). RT-qPCR analysis confirmed the strong induction of *GhFAD2-3* expression under cold conditions (Figure 8). Functional validation through heterologous overexpression in Arabidopsis and virus-induced gene silencing in cotton demonstrated that *GhFAD2-3* positively regulates cold tolerance. Overexpression lines exhibited enhanced cold resistance, whereas VIGS- silenced cotton plants showed increased sensitivity to cold stress (Figure 9 and Figure 10). Physiological analyses revealed that *GhFAD2-3*-weakened plants suffered greater cellular damage than controls, as evidenced by a more pronounced increase in MDA content, coupled with reductions in chlorophyll content. This damage is consistent with oxidative stress caused by elevated levels of reactive oxygen species (ROS) [50]. Accordingly, under cold stress, the activities of the antioxidant enzymes CAT and POD were elevated in *GhFAD2-3*-overexpressing plants but suppressed in *GhFAD2-3*-downregulated cotton plants (Figure 10). Collectively, our findings establish that *GhFAD2-3* contributes to cold stress tolerance in cotton. With the expanding cultivation of upland cotton into northwest inland regions of China where cold stress represents a major constraint, *GhFAD2-3* emerges as a promising genetic target for breeding cold-tolerant cotton varieties.

## 4. Materials and Methods

### 4.1. Identification of FAD Gene Family Members in Gossypium

The genome sequences of *Gossypium hirsutum* acc. TM-1 [41], and its putative diploid ancestors—*G. herbaceum* and *G. arboretum* [51] and *G. raimondii* [52]—were obtained from the CottonGen website [53]. To identify candidate *FAD* genes, a search was conducted against the annotated protein sequences using the FA_desaturase domain (PF00487) from the Pfam database (http://pfam.xfam.org/). This was followed by local BLASTp (v2.17.0) analyses using the amino acid sequences of known Arabidopsis FAD proteins—AtFAD2 (AT3G12120), AtFAD3 (AT2G29980), AtFAD4 (AT4G27030), AtADS3 (AT3G15850), AtFAD6 (AT4G30950), AtFAD7 (AT3G11170), and AtFAD8 (AT5G05580)—as queries. Identified *FAD* genes were named according to their Arabidopsis orthologs and chromosomal locations. The prefixes ‘A’ and ‘D’ were used to denote homologs originating from the At and Dt subgenomes, respectively. The theoretical molecular weight (MW) and isoelectric point (pI) of the *GhFAD* proteins were predicted using ExPASy (accessed on 9 October 2025) [54]. Subcellular localization was predicted using the TargetP-2.0 Server (https://services.healthtech.dtu.dk/services/TargetP-2.0/) (accessed on 9 October 2025). Additionally, a 2 kb genomic region upstream of the initiation codon for each *GhFAD* gene was extracted and submitted to the PlantCARE database (accessed on 15 October 2025) for the identification of cis-regulatory elements in the promoter regions [55].

### 4.2. Phylogenetic, Structural, and Synteny Analysis of FAD Genes

An unrooted phylogenetic tree was constructed using MEGA12 software with the maximum likelihood (ML) method and validated by 1000 bootstrap replicates [56]. The conserved domains within the FAD proteins were identified using the SMART database [57] and visualized with TBtools-II [58]. Furthermore, the ten most conserved protein motifs were identified using the MEME suite with default parameters [59], and their putative functions were annotated via the InterProScan database. Genomic synteny relationships were analyzed using the One Step MCScanX program implemented in TBtools-II, and the resulting synteny blocks were visualized using the Advanced Circos tool within the same software package [60].

### 4.3. Prediction of Transcription Factors and miRNAs Targeting GhFAD Genes

Potential transcription factors (TFs) regulating *GhFAD* genes were predicted using the PlantRegMap database [61], with *G. raimondii* specified as the reference species. To identify miRNAs potentially targeting these genes, the full-length cDNA sequences of *GhFAD* homologs were submitted to the psRNATarget platform (2017 release) for screening against the *G. hirsutum* miRNA database (accessed on 26 October 2025) [62]. The interaction networks among the predicted TFs, miRNAs, and their target *GhFAD* genes were visualized using Cytoscape (v3.10.4) software [63].

### 4.4. Expression Profile Analysis

RNA-Seq datasets encompassing different tissues and multiple stress treatments in *G. hirsutum* var. TM-1 were acquired from the NCBI BioProject under accession number PRJNA490626 [64]. The clean reads from each dataset were aligned to the reference genome using HISAT2, and gene expression levels were then quantified as Fragments Per Kilobase of transcript per Million mapped reads (FPKM) values using StringTie (v2.2.1) software [65].

### 4.5. RNA Extraction and Quantitative Real-Time PCR (RT-qPCR)

Total RNA was extracted from upland cotton and *A. thaliana* plants using the RNAprep Pure Plant Kit (TIANGEN, Beijing, China) according to the manufacturer’s instructions. Following genomic DNA removal, approximately 2 µg of total RNA was reverse-transcribed into first-strand cDNA using the PrimeScript™ RT reagent Kit (TaKaRa, Dalian, China). Gene-specific primers for *GhFAD* genes were designed with Primer5 software based on their coding sequences (Table S1). RT-qPCR was performed as previously described [4], with *GhUBQ7* and *AtActin* genes were serving as an internal control in cotton and *A. thaliana*, respectively (Table S1). Each sample was analyzed with three replicates.

### 4.6. Vector Construction and Arabidopsis Transformation

The full-length coding sequences (CDS) of *GhFAD2-3D* was cloned from 20 days post-anthesis (DPA) embryos of the upland cotton cultivar ZM24. The sequence was subsequently inserted into the pCAMBIA2300 vector using the ClonExpress^®^ MultiS One Step Cloning Kit (Vazyme, Nanjing, China), generating the recombinant construct of 35S::GhFAD2-3. The resulting plasmid was introduced into *Agrobacterium tumefaciens* strain GV3101 via the heat-shock method. *A. thaliana* ecotype Columbia (Col-0) plants were grown in a greenhouse under controlled conditions: 22 °C with a 16/8 h light/dark photoperiod. Transformation was carried out using the floral dip method [66]. Putative transgenic plants were initially screened for kanamycin resistance, and positive transformants were further confirmed by RT-qPCR. T_1_ generation lines exhibiting a 3:1 (positive: negative) segregation ratio (OE-2, OE-3, and OE-4) were selected and considered to harbor a single transgene copy.

### 4.7. Virus-Induced Gene Silencing (VIGS) in Cotton

To investigate the function of *GhFAD2-3* in cotton, a specific 300 bp fragment of *GhFAD2-3D* was cloned into the pTRV2 vector, and the resulting construct was introduced into *A. tumefaciens* strain GV3101. The recombinant agrobacteria were then infiltrated into cotton leaves using a previously described method. Once a photobleaching phenotype was observed in the positive control plants (TRV::GhPDS), leaf samples were collected from the TRV::GhFAD2-3 plants to analyze the target gene expression levels. The experiment included three independent biological replicates, with at least 48 plants analyzed per replicate.

### 4.8. Physiological and Biochemical Indicators Determination

Three-week-old wild-type and *GhFAD2-3*-overexpressing *Arabidopsis* plants were subjected to cold stress at 4 °C for 24 h. In parallel, TRV::00 and TRV::GhFAD2-3 cotton plants at three weeks after infection were exposed to 10 °C for 24 h. Leaves from both *Arabidopsis* and cotton plants were collected before and after cold treatment to assess physiological responses. The parameters measured included malondialdehyde (MDA) and chlorophyll contents, as well as the activities of catalase (CAT) and peroxidase (POD). All biochemical assays were performed using commercial kits (Sinobestbio, Shanghai, China) according to the manufacturer’s instructions. Specifically, MDA content was determined using the MDA Content Assay Kit (A401), chlorophyll content was measured using the Chlorophyll Content Assay Kit (C112), CAT activity was assayed using the Catalase Activity Assay Kit (A501), and POD activity was assayed using the Peroxidase Activity Assay Kit (A502).

## 5. Conclusions

This study presents a comprehensive analysis of the FAD gene family in upland cotton. We systematically investigated the gene structures, conserved domains, and *cis*-acting regulatory elements within promoter regions. Expression profiling revealed distinct spatiotemporal patterns of *GhFAD* genes across various developmental stages and in response to multiple abiotic stresses. Functional characterization further demonstrated the specialized role of *GhFAD2-3* in enhancing cold stress adaptation. While these findings advance our understanding, the precise molecular mechanisms by which FAD genes regulate oil biosynthesis and mediate environmental stress responses remain to be fully elucidated. Additionally, the proposed relay model, involving the sequential action of *GhFAD3-1* and *GhFAD3-2* in fiber development, requires further experimental validation. Nevertheless, this research establishes a solid foundation for the integrated utilization of *FAD* genes in cotton breeding programs, particularly for developing cultivars with improved oil quality, enhanced fiber properties, and greater abiotic stress tolerance.

## Figures and Tables

**Table 1:** Identification and characterization of *GhFAD* genes in *G. hirsutum*. The most probable subcellular localizations are shown for each gene.

Name	Gene Locus ID	CDS	Exons	Amino Acids	Mw (Da)	pI	Subcellular Location
*GhFAD4A*	GhChrA01G0766.1	936	1	311	34,623.59	7.24	mitochondrion
*GhFAD2-4A*	GhChrA01G1558.1	1152	1	383	44,369.41	8.97	peroxisome
*GhFAD2-2A*	GhChrA01G1559.1	1152	1	383	44,367.14	9.06	peroxisome
*GhFAD7/8-1A*	GhChrA01G2801.1	1284	8	427	49,534.07	8.17	plastid
*GhFAD7/8-2A*	GhChrA04G1241.1	1341	8	446	50,961.32	8.47	endoplasmic reticulum
*GhDES6A*	GhChrA05G0444.1	1344	1	447	51,161.89	8.43	plastid
*GhADS3A*	GhChrA05G1321.1	1218	5	405	46,282.05	9.26	endoplasmic reticulum
*GhDES1L-1A*	GhChrA06G1765.1	993	2	330	38,403.38	7.29	chloroplast
*GhFAD3-1A*	GhChrA07G1217.1	1131	8	376	43,504.96	8.84	cytoplasm
*GhSLD2-1A*	GhChrA07G1670.1	1344	1	447	51,183.94	8.36	plastid
*GhSLD2-2A*	GhChrA08G3245.1	1344	1	447	51,201.35	8.64	plastid
*GhFAD3-2A*	GhChrA09G1203.1	1167	8	388	45,033.80	9.05	cytoplasm
*GhDES1L-2A*	GhChrA10G0228.1	996	2	331	38,623.81	8.82	chloroplast
*GhFAD7/8-3A*	GhChrA10G2968.1	1353	8	450	51,185.89	8.91	mitochondrion
*GhSLD2-3A*	GhChrA11G1028.1	1344	1	447	51,379.35	8.94	plastid
*GhFAD2-3A*	GhChrA11G3993.1	1155	1	384	44,247.99	8.96	peroxisome
*GhSLD1A*	GhChrA12G1489.1	1350	2	449	51,874.66	7.98	plastid
*GhFAD3-3A*	GhChrA13G0736.1	927	8	308	35,414.61	8.69	cytoplasm
*GhFAD6A*	GhChrA13G2456.1	1338	10	445	51,585.95	9.09	chloroplast
*GhFAD2-1A*	GhChrA13G2779.1	1158	1	385	44,061.98	9.09	peroxisome
*GhFAD4D*	GhChrD01G0748.1	936	1	311	34,511.51	7.69	chloroplast
*GhFAD2-4D*	GhChrD01G1516.1	1152	1	383	44,283.42	8.94	plastid
*GhFAD2-2D*	GhChrD01G1517.1	1152	1	383	44,184.98	9.04	peroxisome
*GhFAD7/8-1D*	GhChrD01G2692.1	1308	8	435	50,398.01	7.42	plastid
*GhFAD7/8-2D*	GhChrD04G1715.1	1341	8	446	50,716.97	8.52	endoplasmic reticulum
*GhDES6D*	GhChrD05G0455.1	1344	1	447	51,197.00	8.68	plastid e
*GhADS3D*	GhChrD05G1299.1	1161	5	386	44,237.73	9.37	plastid
*GhDES1L-1D*	GhChrD06G1671.1	993	2	330	38,346.32	6.95	plastid
*GhFAD3-1D*	GhChrD07G1186.1	1131	8	376	43,560.10	8.83	plastid
*GhSLD2-1D*	GhChrD07G1624.1	1344	1	447	51,182.96	8.55	plastid
*GhSLD2-2D*	GhChrD08G3126.1	1344	1	447	51,273.35	8.61	cytoplasm
*GhFAD3-2D*	GhChrD09G1113.1	1167	8	388	45,107.92	9.05	cytoplasm
*GhDES1L-2D*	GhChrD10G0246.1	972	2	323	37,794.78	7.30	chloroplast
*GhFAD7/8-3D*	GhChrD10G2859.1	1353	8	450	51,146.85	8.91	mitochondrion
*GhSLD2-3D*	GhChrD11G1055.1	1344	1	447	51,370.34	8.94	plastid
*GhFAD2-3D*	GhChrD11G3903.1	1155	1	384	44,271.03	9.04	peroxisome
*GhSLD1D*	GhChrD12G1413.1	1188	3	395	45,134.87	7.30	plastid
*GhFAD6D*	GhChrD13G2333.1	1329	10	442	51,322.69	9.17	chloroplast
*GhFAD2-1D*	GhChrD13G2655.1	1152	1	383	43,892.69	8.95	endoplasmic reticulum

## Data Availability

The original contributions presented in the study are included in the article/Supplementary Materials, further inquiries can be directed to the corresponding authors.

## Supplementary Materials

The following supporting information can be downloaded at: https://www.mdpi.com/article/10.3390/plants14243767/s1. Table S1. Primers and sequences used in this study. Table S2. Identification and nomenclature of *FAD* genes in *G. herbaceum*, *G. arboreum*, and *G. raimondii*. Table S3. The source data of cis-elements in *GhFAD* promoter regions. Table S4. The source data of regulatory networks of miRNAs targeting *GhFAD* genes. Figure S1. Conserved motifs identified in *GhFAD* proteins. (A) The ten most conserved motifs detected in GhFAD proteins. (B) The logo and sequences of the ten most conserved motifs. Figure S2. Chromosome localization of *GhFAD* members. (A) GhFAD members located on the At subgenome. (B) GhFAD members located on the Dt subgenome. Figure S3. The regulatory relationship of TFs targeting *GhFAD* genes. TFs are depicted as green ovals, and target *GhFAD* genes as blue rectangles.
