# Supplementary figures and images for: Comparative Study of Fatty Acid Desaturase (FAD) Members Reveals Their Differential Roles in Upland Cotton
Source: Plants (Basel). 2025 Dec 10;14(24):3767. doi: 10.3390/plants14243767 (PMC12736523; doi:10.3390/plants14243767)

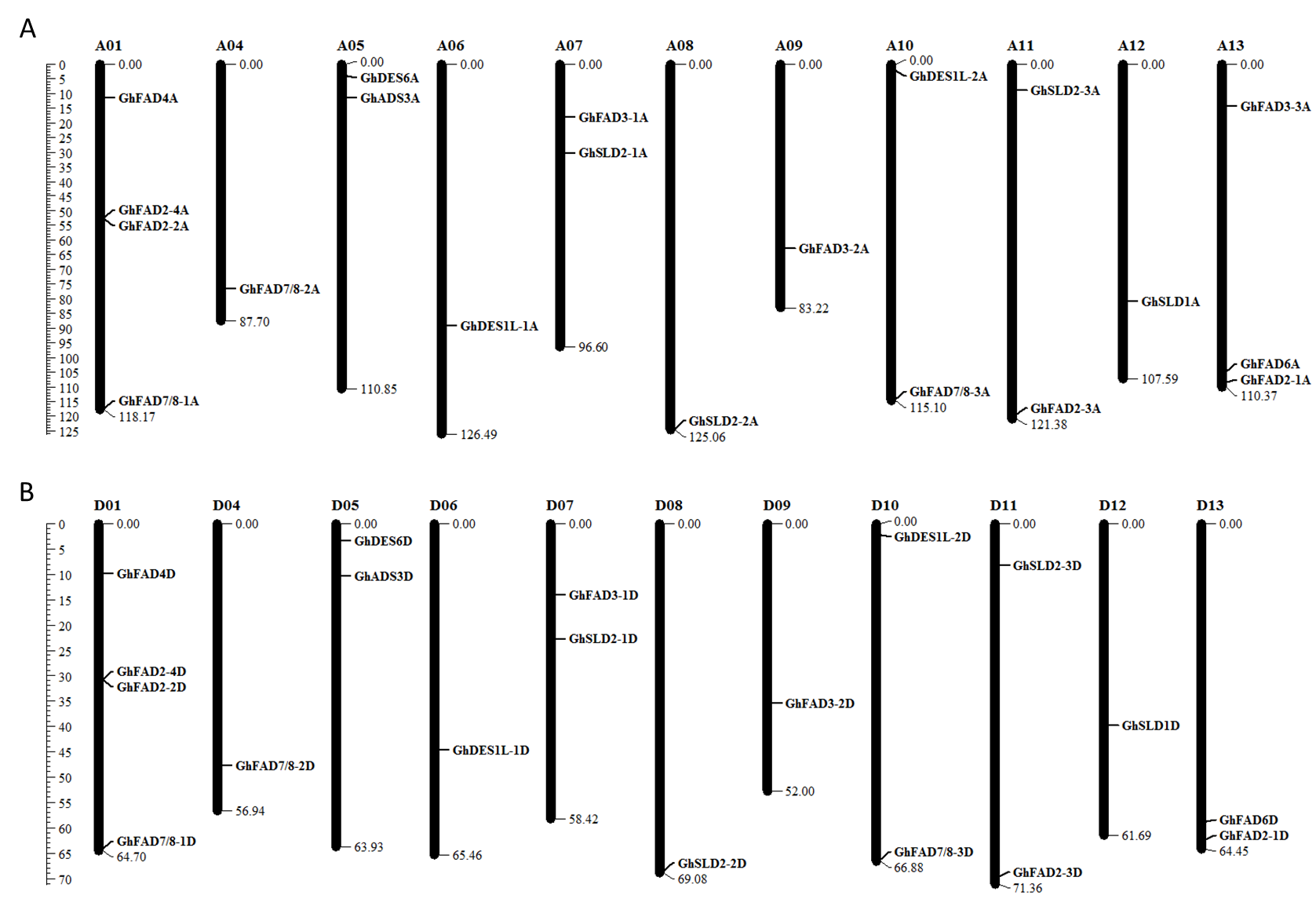

Supplement: Supplementary file 1 [file plants-14-03767-s001.zip › Figure S2.tif]

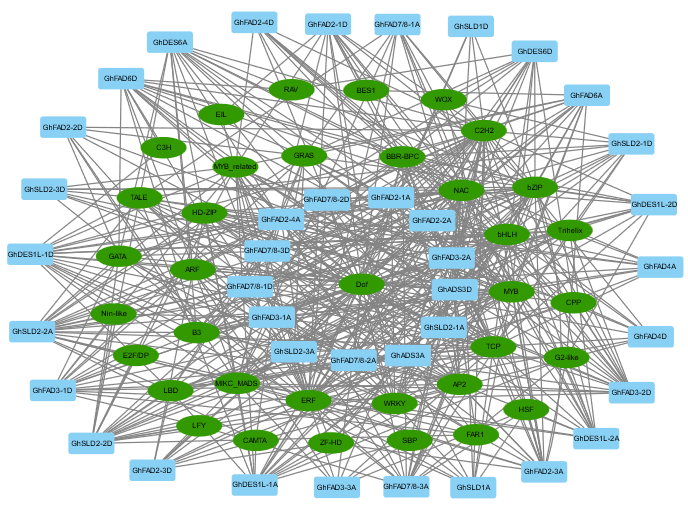

Supplement: Supplementary file 1 [file plants-14-03767-s001.zip › Figure S3.tif]

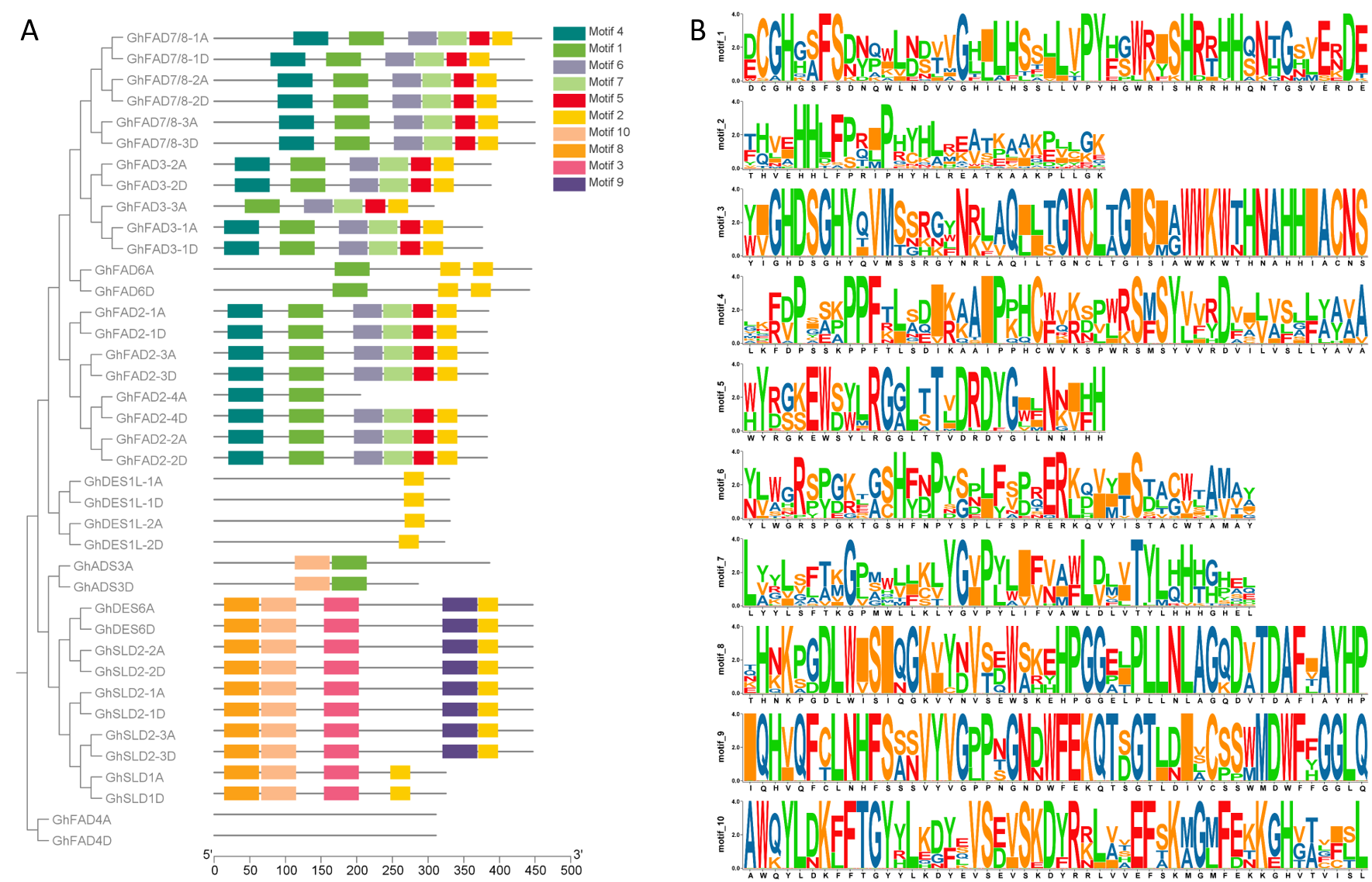

Supplement: Supplementary file 1 [file plants-14-03767-s001.zip › Figure S1.tif]
